# Synthesis of a Novel Zwitterionic Hypercrosslinked Polymer for Highly Efficient Iodine Capture from Water

**DOI:** 10.3390/polym16192846

**Published:** 2024-10-09

**Authors:** Jingwen Yu, Luna Song, Bingying Han, Jiangliang Hu, Zhong Li, Jie Mi

**Affiliations:** 1 State Key Laboratory of Clean and Efficient Coal Utilization, Taiyuan University of Technology, Taiyuan 030024, China; 17836204253@163.com (J.Y.); hanbingying@tyut.edu.cn (B.H.); hujiangliang@tyut.edu.cn (J.H.); 2 Key Laboratory of Coal Science and Technology, Ministry of Education, Taiyuan University of Technology, Taiyuan 030024, China; 3 Lu’an Chemical Group Co., Ltd., Changzhi 046204, China; 4 Shanxi Institute of Energy, Jinzhong 030600, China; sxdxsln@163.com

**Keywords:** zwitterionic hypercrosslinked polymer, iodine, aqueous solution, adsorption

## Abstract

Cationic porous organic polymers have a unique advantage in removing radioactive iodine from the aqueous phase because iodine molecules exist mainly in the form of iodine-containing anions. However, halogen anions will inevitably be released into water during the ion-exchange process. Herein, we reported a novel and easy-to-construct zwitterionic hypercrosslinked polymer (7AIn-PiP)-containing cationic pyridinium-type group, uncharged pyridine-type group, pyrrole-type group, and even an electron-rich phenyl group, which in synergy effectively removed 94.2% (456 nm) of I_2_ from saturated I_2_ aqueous solution within 30 min, surpassing many reported iodine adsorbents. Moreover, an I_2_ adsorption efficiency of ~95% can still be achieved after three cyclic evaluations, indicating a good recycling performance. More importantly, a unique dual 1,3-dipole was obtained and characterized by ^1^H/^13^C NMR, HRMS, and FTIR, correlating with the structure of 7AIn-PiP. In addition, the analysis of adsorption kinetics and the characterization of I_2_@7AIn-PiP indicate that the multiple binding sites simultaneously contribute to the high affinity towards iodine species by both physisorption and chemisorption. Furthermore, an interesting phenomenon of inducing the formation of HIO_2_ in unsaturated I_2_ aqueous solution was discovered and explained. Overall, this work is of great significance for both material and radiation protection science.

## 1. Introduction

On 24 August 2023, the Fukushima Daiichi nuclear power plant began discharging more than 1.3 million tons of nuclear sewage into the Pacific Ocean, and will continue this behavior over the next three decades [1,2]. Japan’s nuclear sewage contains many kinds of harmful radionuclides, among which the excessive amount of ^129^I and ^131^I has aroused social concern because they can significantly induce thyroid cancer [3,4]. In particular, ^129^I has an ultra-long half-life (~1.57 × 10^7^ years) [5,6]. If not appropriately handled, the hazardous radioiodine will inevitably have a serious impact on the marine ecosystem and cause great harm to human health. Therefore, it is necessary and meaningful to delve the removal of radioactive iodine in water. The solid-phase adsorption method is considered as an effective way to remove radioiodine from water due to its low cost, high efficiency, simple operation, and its use as a recyclable and reusable adsorbent [7].

As an important subgroup of porous organic polymers (POPs), ionic porous organic polymers (i-POPs) are novel and fascinating porous organic materials in which ionic moieties can be directly incorporated or post-synthetically added to the polymer backbones or side chains [8,9]. The high charge density and strong polarizability of i-POPs endow them with unique advantages in electrostatic interaction applications [10]. The recent literature reflects an upsurge in the development of i-POPs such as electrospun fiber adsorbents (N-MOF-PAN fibers) [11], imidazolium-based i-POP [12], and benzoquinone-derived porous hydrophenazine frameworks [13] for iodine removal from water. As is known to all, iodine molecules (I_2_) tend to form triiodide anions (I_3_^−^), which are the major components in aqueous phases [12,14,15]. All these works found that cationic binding sites are beneficial for enriching I_3_^−^ through electrostatic interactions driving the ion-exchange process from Br^−^/Cl^−^ to I_3_^−^ [11,12,13].

With the development of the synthetic methodology, diverse ionic building blocks and i-POPs with unique structures can be rationally designed. In fact, the ionization of organic skeletons is a common phenomenon. For example, the N atoms in aromatic heterocyclic/aliphatic compounds are easily ionized through S_N_2 nucleophilic substitution reactions [11,12] or protonated in an acidic solution [13], which leads to quaternization and generates cationic frameworks. It should be noted that the negative halogen ions are simultaneously introduced into the frameworks of the corresponding polymers as equilibrium ions. Although the application of i-POPs for iodine capture from water has made some progress, it is still limited due to the additional negative halogen ions that are released into the aqueous phase during the adsorption process. To solve this issue, the design and development of novel i-POPs for iodine capture without the ion-exchange process in aqueous solution remain urgent tasks. 

Inspired by the one-pot synthesis of viologen-based hypercrosslinked polymers via a sequential process of ionization and polymerization [16], herein, a similar strategy was applied to construct a novel i-POP with the zwitterionic scaffold. 7-azaindole (7AIn) and the excess α,α-dichloro-*p*-xylene (DCX) were selected as building blocks. Initially, the zwitterionic building unit was predesigned via S_N_2 nucleophilic substitution and the pyrrole-type H elimination reactions between 7AIn and DCX. Subsequently, Friedel–Crafts alkylation reaction was used to construct a hypercrosslinked network under the catalysis of anhydrous FeCl_3_. More importantly, the formation mechanism of the obtained polymer (7AIn-PiP) was proposed by analyzing the structure of the important intermediate product. To the best of our knowledge, a zwitterionic hypercrosslinked polymer for iodine adsorption from water has rarely been reported. In this paper, the utility of 7AIn-PiP for highly efficient iodine capture from water was also demonstrated. The adsorption kinetics and the recycling performance of 7AIn-PiP from saturated I_2_ aqueous solution were evaluated systematically. Furthermore, the iodine adsorption mechanism was comprehensively described by analyzing the characterizations of the iodine-loaded 7AIn-PiP. Ultimately, a new phenomenon of iodine adsorption by 7AIn-PiP in unsaturated I_2_ aqueous solution was discovered and explained.

## 2. Experimental Section

### 2.1. General Remarks

#### 2.1.1. Materials

All reagents used in this work were purchased from commercial sources without further purification unless otherwise stated. 1,2-dichloroethane (DCE) was dehydrated with CaH_2_ prior to use. Nonradioactive I_2_ was used in iodine adsorption experiments. 

#### 2.1.2. Characterization

N_2_ adsorption–desorption tests were performed on a JW-BK122W N_2_-sorption apparatus (Beijing JWGB Sci. & Tech. Co., Ltd., Beijing, China); the specific surface area of the sample was calculated based on the Bruner–Emmett–Teller (BET) equation, and the pore size distribution curve was obtained based on BJH theory. UV–Vis absorption spectra were tested on a spectrophotometer (Varian Cary 300, Palo Alto, CA, USA). Fourier-transformed infrared (FTIR) spectra were conducted on a Bruker Vertex 70 FTIR instrument, Billerica, MA, USA (KBr tablet method). ^1^H NMR and ^13^C NMR spectra were measured on a JEOL 600 MHz spectrometer using H_2_O and DMSO as the internal standards in DMSO-*d*_6_. High-resolution mass spectroscopies (HRMSs) were recorded using a Thermo Scientific Q Exactive mass spectrometer, Waltham, MA, USA. Solid-state ^13^C cross-polarization/magic-angle-spinning (CP/MAS) nuclear magnetic resonance (NMR) spectra were recorded using a Bruker Avance III 600 MHz NMR spectrometer. Electron paramagnetic resonance (EPR) spectroscopy was observed using a Bruker EMXplus-9.5/12 spectrometer. Scanning electron microscopy (SEM) images were obtained by using a Hitachi S-4800 field emission scanning electron microscope, Tokyo, Japan. Transmission electron microscopy (TEM) images were recorded on a JEOL JEM-2100F transmission electron microscope with an acceleration voltage of 200 kV. Powder X-ray diffraction (PXRD) patterns were collected on a Shimadzu XRD-6000 X-ray powder diffractometer, Kyoto, Japan, with Cu Kα radiation. X-ray photoelectron spectroscopy (XPS) was measured using a Thermo Scientific K-Alpha X-ray photoelectron spectrometer. Raman spectroscopy was measured on a Horiba LabRAM HR Evolution Raman spectrometer, Kyoto, Japan (laser wavelength: 514 nm).

### 2.2. Synthesis of Materials

The detailed descriptions about the “preparation and characterization of I_2_ aqueous solutions” and “preparation of a batch of polymers and screening of the optimal adsorbent” are provided in Supplementary Materials Sections S1 and S2, respectively.

#### 2.2.1. Synthesis of 7AIn-PiP

**Scheme 1:**
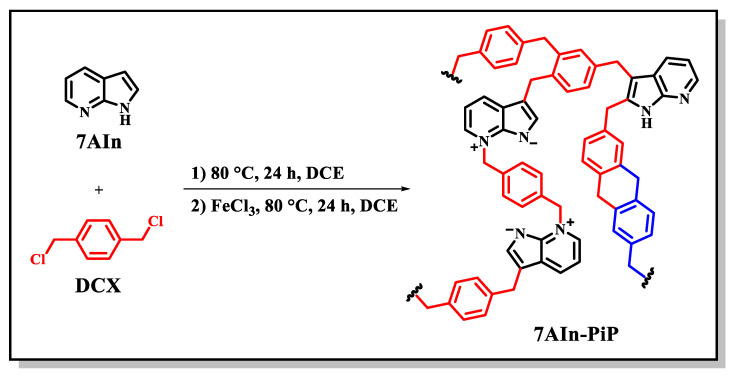
Schematic representation for the synthesis of 7AIn-PiP.

Under N_2_ protection, 7AIn (295.4 mg, 2.5 mmol) and DCX (1.7506 g, 10 mmol) were added into a dry 100 mL double-necked round-bottomed flask equipped with a reflux condenser and a magnetic stirrer. After DCE (60 mL) was injected into the flask, the mixture was stirred at 80 ℃ for 24 h. After the reaction system was cooled to room temperature, anhydrous FeCl_3_ (1.6220 g, 10 mmol) was added to the mixture under N_2_ protection as soon as possible. Then, the obtained mixture was refluxed at 80 °C for an additional 24 h. After being cooled down to room temperature again, the insoluble solid was obtained by suction filtration and continuously washed with methanol to remove the unreacted monomers, oligomers, and FeCl_3_ until the filtrate became colorless. The crude product was further extracted with methanol by Soxhlet extraction for 24 h, and the corresponding yellow-brown powder (1.2483 g, 95%) was obtained by vacuum drying at 90 °C for another 24 h. The schematic representation for the synthesis of 7AIn-PiP is illustrated in Scheme 1. 

#### 2.2.2. Synthesis of the Dual 1,3-Dipole

In order to precisely unveil the composition and structural information of 7AIn-PiP, we strived to obtain the new product after the S_N_2 nucleophilic substitution reaction. 

**Scheme 2:**
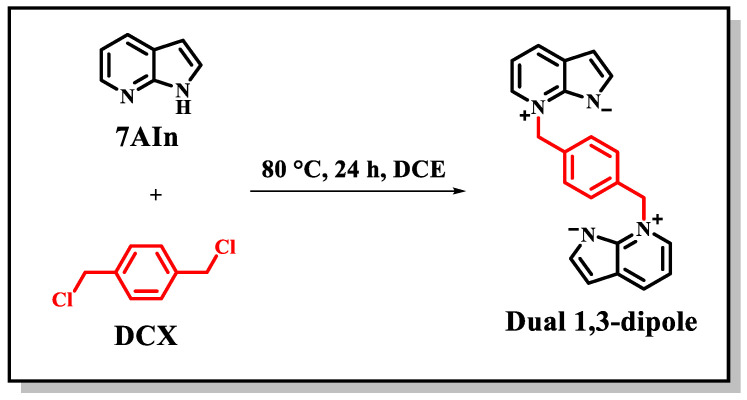
Schematic representation for the synthesis of the dual 1,3-dipole.

Under a N_2_ atmosphere, 7AIn (295.4 mg, 2.5 mmol) and DCX (1.7506 g, 10 mmol) were added into a dry 100 mL double-necked round-bottomed flask equipped with a reflux condenser and a magnetic stirrer. Then, 60 mL of DCE was injected into the flask and the mixture was stirred at 80 °C for 24 h. After being cooled down to ambient temperature, an appropriate amount of silica gel was added into the solution, and the solvent was removed under reduced pressure. The obtained mixture was loaded and purified through silica gel column chromatography with gradient eluent (ethyl acetate:petroleum ether = 1:4 (*v*/*v*), then methanol). After evaporation of the eluate under reduced pressure, 7AIn (120.5 mg) and DCX (1.5750 g) that did not participate in the reaction were recovered. Most importantly, a new white solid product with a yield of 235.5 mg was obtained. The schematic representation for the synthesis of the dual 1,3-dipole is depicted in Scheme 2. 

### 2.3. I_2_ Adsorption Experiments of 7AIn-PiP in I_2_ Aqueous Solution

#### 2.3.1. I_2_ Adsorption Experiments of 7AIn-PiP in Saturated I_2_ Aqueous Solution

The detailed information about the “adsorption kinetics of 7AIn-PiP in saturated I_2_ aqueous solution” is provided in Supplementary Materials Section S3. To further investigate the I_2_ adsorption mechanism of 7AIn-PiP in saturated I_2_ aqueous solution, the resulting mixture was filtered under ambient pressure when the saturation adsorption was reached, and the filter cake was dried in open air to obtain the iodine-loaded 7AIn-PiP (named as I_2_@7AIn-PiP), which was characterized with XPS, Raman, and FTIR spectra to confirm the main forms of iodine in the polymer skeleton.

#### 2.3.2. Recycling Tests of 7AIn-PiP in Saturated I_2_ Aqueous Solution

To evaluate the reusability of 7AIn-PiP, I_2_@7AIn-PiP was extracted with ethanol by Soxhlet extraction for 48 h (fresh ethanol was replaced every 12 h), and the obtained sample was vacuum-dried at 90 °C for 24 h, reusing for the next round of adsorption experiment. The cyclic tests were performed three times.

#### 2.3.3. I_2_ Adsorption Experiments of 7AIn-PiP in Unsaturated I_2_ Aqueous Solution

Firstly, 126.9 mg of I_2_ was completely dissolved in 500.0 mL of deionized water to prepare 1.0 mmol L^−1^ of I_2_ aqueous solution. Next, 50.0 and 10.0 mL of stock solutions were diluted to a volume of 100 mL by deionized water to obtain I_2_ aqueous solutions with the concentrations of 0.5 and 0.1 mmol L^−1^, respectively. The UV–Vis spectra of these three I_2_ aqueous solutions were determined. Finally, at room temperature, 5.0 mg of 7AIn-PiP was added to 10 mL of I_2_ aqueous solution with different concentrations (1.0, 0.5, and 0.1 mmol L^−1^) and stirred at a speed of 500 rpm. The UV–Vis spectra of these solutions were recorded after 30 min.

## 3. Results and Discussion

### 3.1. Characterization and Analysis of Materials

#### 3.1.1. Pore Properties and I_2_ Adsorption Results of a Batch of Polymers

As indicated in Supplementary Materials Section S2, it was found that using DCX can increase specific surface areas and total pore volume. The existence of benzyl cross-linked bridges and the extended structures resulting from the self-condensation of DCX might prevent the ionic building blocks reacting directly with each other. The more DCX is used, the larger the specific surface area and total pore volume of the corresponding polymer. With the increase in DCX/7AIn molar ratio, the specific surface area increased from 14 to 814 m^2^ g^−1^. If the molar ratio of 7AIn to DCX was 1:2, Polymer-1 displayed the lowest specific surface area and total pore volume because the charge interaction and low degree of crosslinking mean that intermolecular packing easily occurred among the charged intermediate products [9]. If no 7AIn was added, the self-polymerization of DCX endowed Polymer-6 with the largest specific surface area and total pore volume. However, neither Polymer-1 nor Polymer-6 showed a satisfactory I_2_ adsorption performance. Notably, Polymer-2 exhibited the highest I_2_ removal rate among these polymers, indicating that the 7AIn/DCX molar ratio of 1:4 is the optimum proportion, as lower or higher molar ratio resulted in poor adsorption properties. In what follows, we renamed Polymer-2 as 7AIn-PiP. The above results demonstrate that the I_2_ removal rate is determined by both the nitrogen-containing affinity site and high specific surface area.

#### 3.1.2. Characterization of 7AIn-PiP

**Figure 1:**
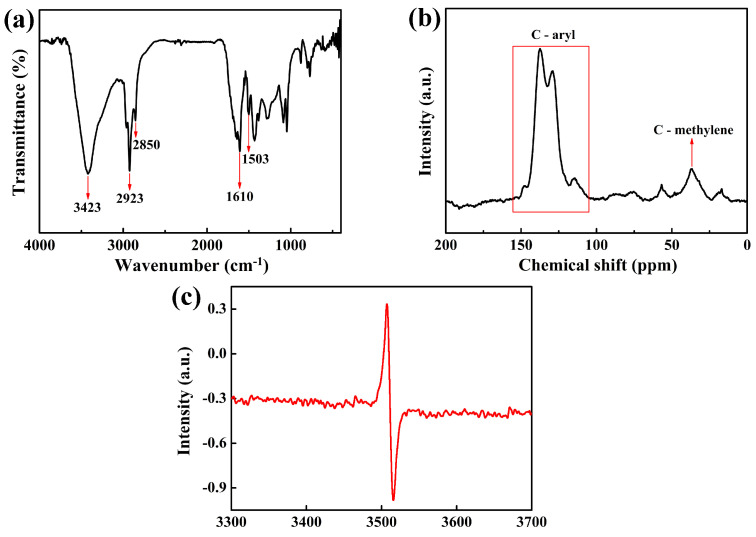
(**a**) FTIR, (**b**) ^13^C CP/MAS NMR (the red rectangle is the region of aromatic carbon), and (**c**) EPR spectra of 7Ain-PiP.

In order to identify the functional groups on 7AIn-PiP, FTIR and ^13^C CP/MAS NMR spectra were performed. Figure 1a shows that the signal at about 3423 cm^−1^ corresponds to N–H stretching vibration on the 7AIn ring, which may be overlapped by the signal of O–H stretching vibration of water. The peaks at around 2923 and 2850 cm^−1^ can be ascribed to the C–H stretching vibration of the methylene group. The bands at approximately 1610 and 1503 cm^−1^ are attributed to the stretching vibration of aromatic rings (7AIn ring and benzene ring). In addition, as shown in Figure 1b, the broad peaks in the region of 155–105 ppm correspond to the aromatic carbon, while the methylene carbon originating from DCX is located at around 37 ppm. According to the above analysis, both FTIR and ^13^C CP/MAS NMR spectra demonstrate that 7AIn reacts efficiently with DCX to incorporate a certain amount of the methylene group through Friedel–Crafts alkylation reaction, confirming the successful preparation of 7AIn-PiP. Furthermore, the appearance of a strong paramagnetic signal in the ESR spectrum (Figure 1c) reveals that charges actually exist in the skeleton of 7AIn-PiP.

**Figure 2:**
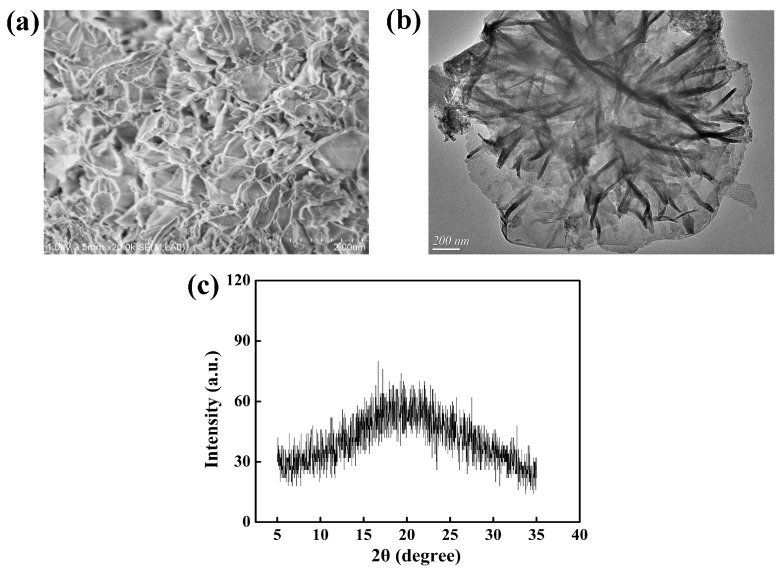
(**a**) SEM (scale bar: 2 μm) image, (**b**) TEM (scale bar: 200 nm) image, and (**c**) PXRD pattern of 7Ain-PiP.

The morphology of powdered 7AIn-PiP was investigated by SEM and TEM. The observed images reveal that 7AIn-PiP exhibits an irregular wrinkled morphology. The porous spaces were formed by the agglomeration of the membrane-like structures (Figure 2a,b). To further demonstrate the crystallinity of 7AIn-PiP, PXRD was conducted. As shown in Figure 2c, the diffraction peak in the pattern is not obvious, but is relatively broad, indicating that 7AIn-PiP displays an amorphous phase without long-range order, which is consistent with the results of SEM and TEM. 

**Figure 3:**
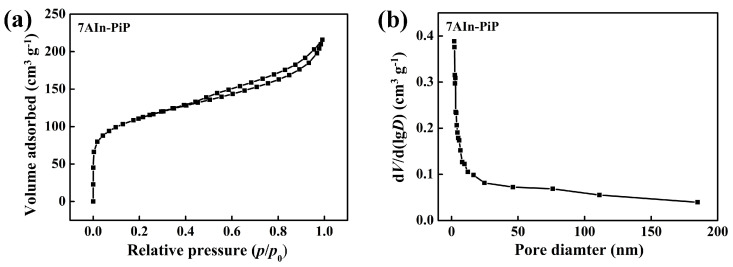
(**a**) N_2_ adsorption-desorption isotherms at 77 K and (**b**) pore size distribution curve calculated by BJH theory.

The pore property of 7AIn-PiP was characterized by using a standard N_2_ adsorption–desorption experiment at 77 K up to 1 bar (sample was activated under a vacuum at 120 °C for 12 h). According to the International Union of Pure and Applied Chemistry (IUPAC) classification of physical adsorption isotherms, 7AIn-PiP displays a mixture of type I and type II N_2_ adsorption isotherms with a type H4 hysteresis loop (Figure 3a), suggesting that 7AIn-PiP mainly contains micropores and mesopores. Moreover, the obtained pore size distribution curve (Figure 3b) for 7AIn-PiP exhibits abundant microporosity and a partial mesoporous character, which is in accordance with the sharp rise curve and mild capillary condensation in the isotherms. 

#### 3.1.3. Characterization of the Dual 1,3-Dipole

**Figure 4:**
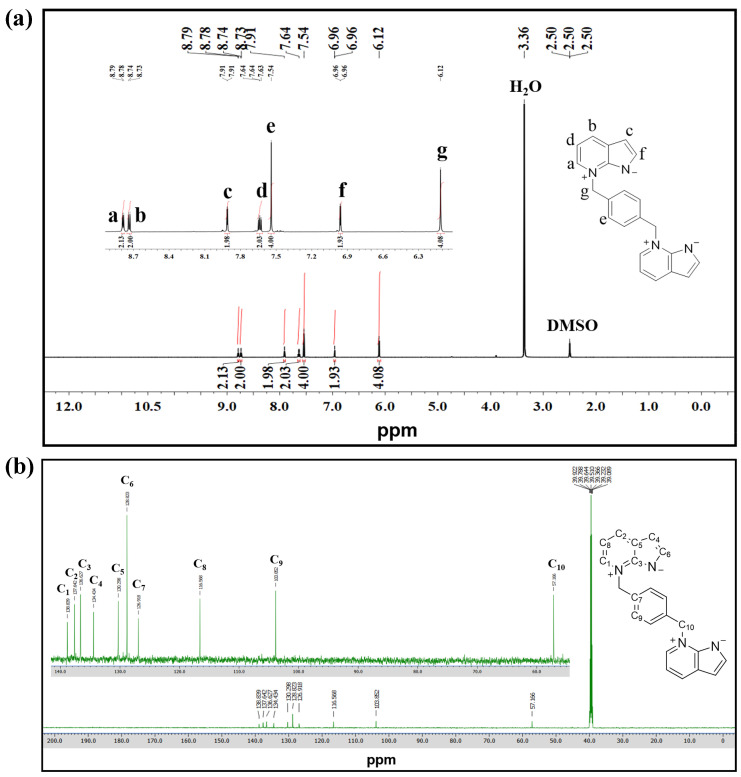
(**a**) ^1^H NMR (the letters in the picture represent different types of H atoms) and (**b**) ^13^C NMR spectra of the dual 1,3-dipole.

In order to confirm the structure of the new obtained product after the S_N_2 nucleophilic substitution reaction, ^1^H NMR (Figure 4a), ^13^C NMR (Figure 4b), and FTIR (Supplementary Materials Section S4) were performed. It is worth noting that there is no signal of H atoms attached to the pyrrole-type N in the ^1^H NMR spectrum. Instead, the characteristic peaks in the FTIR spectrum and the number and types of H/C atoms in the ^1^H/^13^C NMR spectra could be matched with an interesting dual 1,3-dipole. The relevant data of liquid ^1^H NMR and ^13^C NMR are presented as follows: ^1^H NMR (600 MHz, DMSO-*d*_6_) *δ*_ppm_: 8.79 (d, *J* = 6.0 Hz, 2H), 8.74 (d, *J* = 7.8 Hz, 2H), 7.91 (d, *J* = 3.6 Hz, 2H), 7.64 (dd, *J* = 7.8; 6.0 Hz, 2H), 7.54 (s, 4H), 6.96 (d, *J* = 3.6 Hz, 2H), 6.12 (s, 4H). Calibration (H_2_O-3.36 ppm, DMSO-2.50 ppm). ^13^C NMR (150 MHz, DMSO-*d*_6_) *δ*_ppm_: 138.84 (C_1_), 137.64 (C_2_), 136.63 (C_3_), 134.43 (C_4_), 130.30 (C_5_), 128.82 (C_6_), 126.92 (C_7_), 116.57 (C_8_), 103.85 (C_9_), 57.17 (C_10_). Calibration (DMSO-39.51 ppm). 

**Figure 5:**
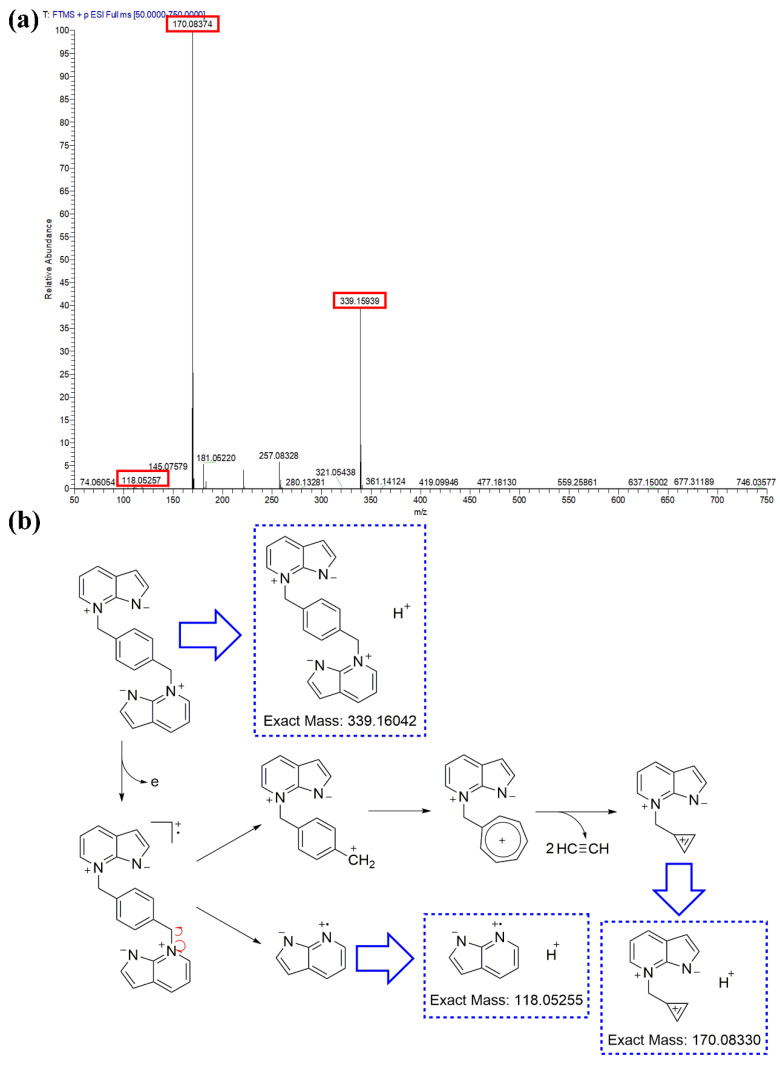
(**a**) HRMS spectrum (the red rectangles represent the characteristic peaks that need to be focused) and (**b**) the mass spectrometry fragmentation pathway of the dual 1,3-dipole.

As shown in Figure 5a, HRMS further confirms the accuracy of molecular weight: calcd for [M + H]^+^ *m*/*z* = 339.16042, found *m*/*z* = 339.15939. The characteristic fragmentation pathway of the dual 1,3-dipole is shown in Figure 5b. Initially, the dual 1,3-dipole receives energy from the ion source, loses an electron, and thus becomes a positively charged molecular ion. Subsequently, the resulting molecular ion undergoes the homolytic cleavage of benzyl C–N bond (α-cleavage), forming a benzylic carbocation. Lastly, the obtained benzylic carbocation immediately transforms into a more stable tropyllium ion and further loses two acetylene molecules, producing a cyclopropenyl cation. In the positive-ion mode, the strongest peak (base peak, relative abundance 100%) with a mass-to-charge ratio (*m*/*z*) of 170.08374 [M + 1] and the molecular ion peak with a mass-to-charge ratio (*m*/*z*) of 339.15939 [M + 1] obviously appear in Figure 5a. Meanwhile, it is worth noting that there is also a weak peak with a mass-to-charge ratio (*m*/*z*) of 118.05257 [M + 1] in the spectrum, which is the value of the other part of the molecular ion after the α-cleavage. These results demonstrate the rationality of the fragmentation pathway and the accuracy of the structure of the dual 1,3-dipole.

#### 3.1.4. Formation Mechanism of the Dual 1,3-Dipole and Polymer 7AIn-PiP

**Scheme 3:**
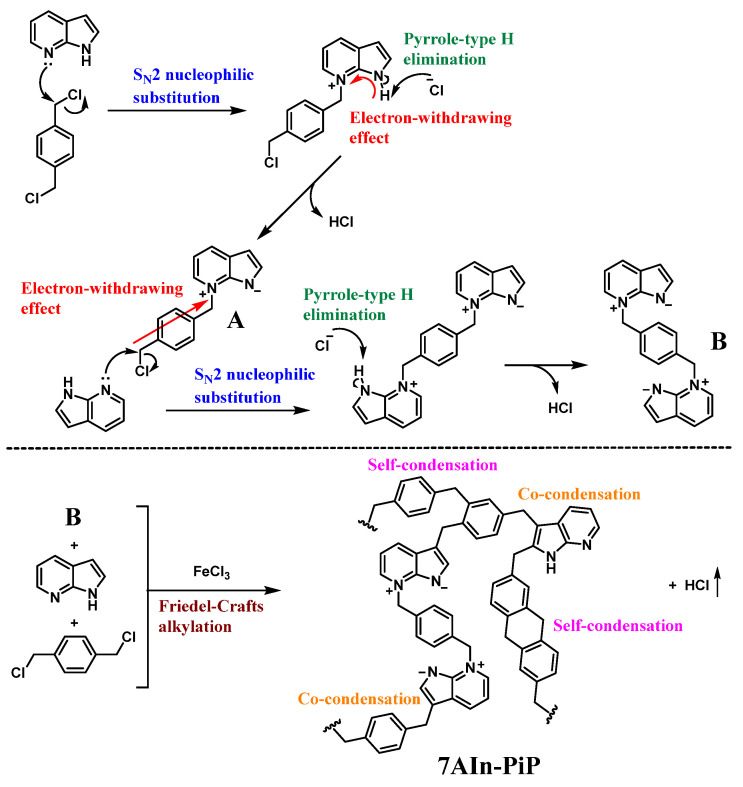
Schematic diagram of formation mechanism of the dual 1,3-dipole and polymer 7AIn-PiP.

Based on the above structural information, a possible formation mechanism of the dual 1,3-dipole and 7AIn-PiP is proposed. As illustrated in Scheme 3, in the first stage, the pyridine-type N of 7AIn attacks the benzyl on one side of DCX, forming an ionic C–N covalent bond (quaternization reaction) [17,18,19,20]. By virtue of the formation of the pyridinium-type chloride salt, the electron-withdrawing ability of the positively charged pyridinium-type N^+^ is stronger than that of electroneutral pyridine-type N, which effectively weakens the electron cloud density between the N–H bond of the pyrrole-type moiety. Accordingly, the H atom attached to the pyrrole-type N is easily captured by the Cl^−^ to form the by-product HCl, which escapes from the reaction system under the heating condition. It is notable that 1,3-dipole **A** was not obtained by column chromatography because the strong electron-withdrawing ability of the positively charged pyridinium-type N^+^ makes a difference once again; the electron cloud density between the C–Cl bond of 1,3-dipole **A** is lower than that of the DCX molecule, meaning that the Cl atom on 1,3-dipole **A** is more easily replaced by another 7AIn molecule. After going through a similar process (S_N_2 nucleophilic substitution and pyrrole-type H elimination reactions) as described above, the dual 1,3-dipole **B** is generated. It is also worth noting that not all 7AIn molecules participate in S_N_2 nucleophilic substitution reactions due to the limitation of its own structure; the pyrrole-type moiety has a certain steric hindrance effect on the nucleophilic attack of the pyridine-type moiety. In the second stage, a zwitterionic hypercrosslinked organic network (7AIn-PiP) with permanent porosity can be formed by a one-pot FeCl_3_-promoted Friedel–Crafts alkylation reaction, including the co-condensation among the dual 1,3-dipole **B** (ionic building unit), residual 7AIn (electroneutral building unit), and the excess DCX (crosslinker), in addition to the self-condensation of DCX. Meanwhile, the by-product HCl is still released from the reaction system under the heating condition.

### 3.2. I_2_ Adsorption Property of 7AIn-PiP

#### 3.2.1. Evaluation of I_2_ Adsorption Property of 7AIn-PiP in Saturated I_2_ Aqueous Solution

**Figure 6:**
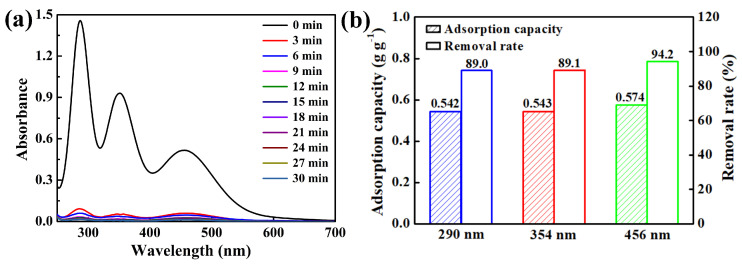
(**a**) Time-dependent UV-Vis absorption spectra of saturated I_2_ aqueous solution (1.2 mmol L^−1^, 10.0 mL) upon adding 5.0 mg of 7AIn-PiP. (**b**) Corresponding I_2_ adsorption capacities and I_2_ removal rates at adsorption equilibrium for 7AIn-PiP.

The time-dependent UV–Vis absorption spectra of I_2_ aqueous solution upon adding 7AIn-PiP are shown in Figure 6a. The capture of iodine species by 7AIn-PiP can achieve adsorption equilibrium within 30 min. The corresponding I_2_ saturation adsorption capacities and I_2_ removal rates at different characteristic absorption wavelengths are presented in Figure 6b. In order to assess the I_2_ adsorption property of 7AIn-PiP in saturated I_2_ aqueous solution, the I_2_ removal rates of recently reported materials under the similar test conditions are summarized in Supplementary Materials Section S5. Benefitting from the integration of the abundant adsorption sites and pore structures, 7AIn-PiP demonstrates a high I_2_ adsorption efficiency from water.

#### 3.2.2. Analysis of Adsorption Kinetics of 7AIn-PiP in Saturated I_2_ Aqueous Solution 

**Figure 7:**
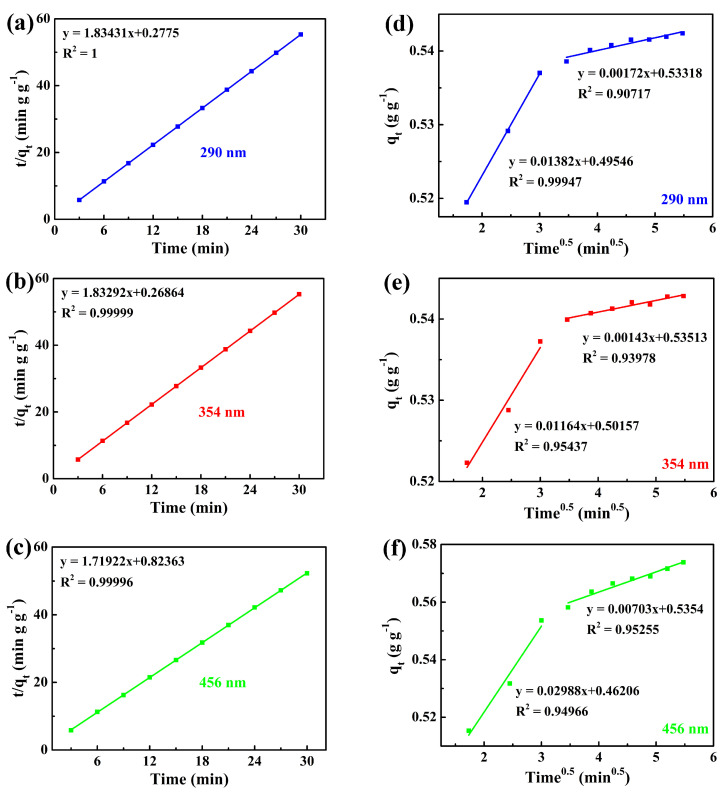
Fitting plots of I_2_ adsorption data with (**a**–**c**) pseudo-second-order and (**d**–**f**) intra-particle diffusion kinetic models, respectively.

Initially, we used the pseudo-second-order kinetic model to fit the adsorption data. The corresponding fitting plots and correlated parameters based on this model are shown in Figure 7a–c and Table 1. The correlation coefficients (*R*^2^) are close to 1, and the experimental values (*q*_e,exp_) are close to the calculated adsorption capacities (*q*_e,cal_) at different characteristic absorption wavelengths, implying that the whole adsorption process of 7AIn-PiP can be reliably revealed by the pseudo-second-order kinetic model with a good fitting effect. The pseudo-second-order kinetic model assumes that the adsorption process is controlled by chemisorption [21], but a good fitting result does not mean that the I_2_ adsorption process is completely driven by chemisorption [22,23], which may be affected by both chemisorption and physisorption. 

The pseudo-second-order kinetic model is not suitable for explaining the diffusion (mass transfer) process of solute on the adsorbent. In order to further describe the actual rate-controlling step, the intra-particle diffusion kinetic model was also used to fit the adsorption data. As can be seen from Figure 7d–f and Table 2, the intercepts of the linear equations of all fitting curves are not 0, implying that the adsorption rate is influenced by both liquid-film diffusion and intra-particle diffusion. All fitting curves at different characteristic absorption wavelengths can be divided into two stages. In the initial stage of the adsorption process, the slopes of the curves are large, denoting that the adsorption rate is fast. There is a large concentration gradient between the surface of 7AIn-PiP and the solute in the solution, thus providing a large driving force of mass transfer. The solutes rapidly diffuse to the outer surface of 7AIn-PiP and are adsorbed by the adsorption sites. The adsorption amount increases with the extension of time. However, in the later stage of the adsorption process, the curves have small slopes, signifying that the adsorption rate decreases. The solutes further enter the internal pores of 7AIn-PiP and are absorbed by the adsorption sites on the inner surface of the pores. When the adsorption sites are occupied and the concentration of the solute decreases, the adsorption process gradually reaches the equilibrium state.

#### 3.2.3. Characterization of I_2_@7AIn-PiP

**Figure 8:**
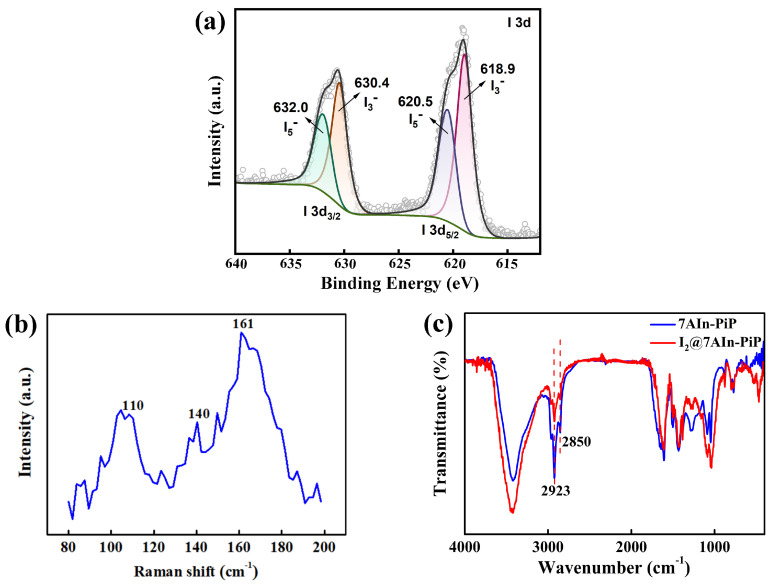
(**a**) XPS and (**b**) Raman spectra of I_2_@7AIn-PiP. (**c**) FTIR spectra of 7AIn-PiP and I_2_@7AIn-PiP.

As shown in the XPS spectrum of I_2_@7AIn-PiP (Figure 8a), two types of polyiodide peaks I_3_^−^ (618.9 and 630.4 eV) and I_5_^−^ (620.5 and 632.0 eV) generated by the splitting of I 3d_3/2_ and I 3d_5/2_ orbital levels [11,24,25,26,27] can be observed. Moreover, the species of iodine within 7AIn-PiP were also confirmed by Raman spectrum (Figure 8b). Three characteristic peaks at 110, 140, and 161 cm^−1^ can be assigned to the vibration modes of polyiodides, mainly I_3_^−^ and I_5_^−^ anions. Of these, the bands at 110 and 140 cm^−1^ are attributed to the symmetric and asymmetric stretching vibrations of I_3_^−^, respectively, while the peak at 161 cm^−1^ is assigned to the stretching vibration of I_5_^−^ [28,29,30,31] (consistent with I_5_^−^ in the L or V configuration [32]). The results of XPS and Raman spectra demonstrate that I_3_^−^ and I_5_^−^ coexist inside the framework of 7AIn-PiP. Furthermore, comparing the FTIR spectra of 7AIn-PiP and I_2_@7AIn-PiP (Figure 8c) reveals that the characteristic bands at 2923 and 2850 cm^−1^ attributed to the C–H stretching vibration of the methylene group weaken significantly after adsorption, providing further evidence of the interaction between the guest iodine species and the host polymer skeleton.

#### 3.2.4. Possible I_2_ Adsorption Mechanism of 7AIn-PiP in Saturated I_2_ Aqueous Solution

**Scheme 4:**
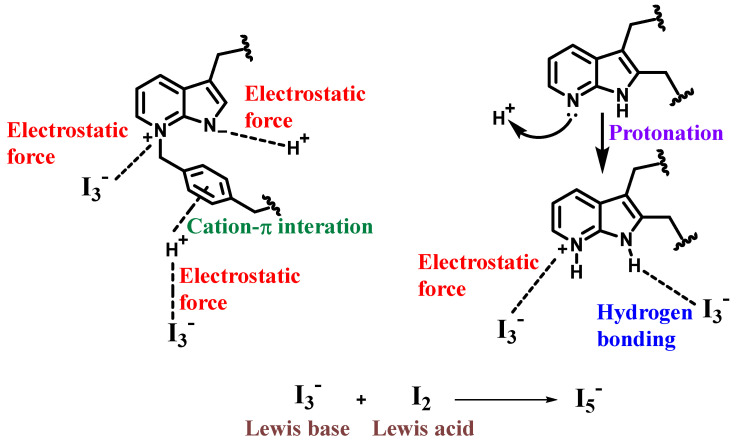
Schematic diagram of the possible I_2_ adsorption mechanism of 7AIn-PiP in saturated I_2_ aqueous solution.

According to the above analysis, the possible I_2_ adsorption mechanism of 7AIn-PiP from water can be proposed as follows. The major component I_3_^−^ can interact with three kinds of cationic binding sites: (1) I_3_^−^ can be directly adsorbed through electrostatic force, which comes from the available cationic pyridinium-type group in the zwitterionic skeleton [11,12,13]; meanwhile, H^+^ in the solution can be captured by the pyrrole-type N^−^ site. (2) It is well known that the pyridine-type N is easily protonated in an acidic environment [33,34]. After protonation, the pyridine-type group could be converted into another kind of cationic pyridinium-type group. Then, the new obtained cationic binding site also adsorbs I_3_^−^ by electrostatic force; (3) the electron-rich benzene ring from DCX as another functional site interacts with H^+^ through cation–π interaction [35,36], forming cationic–π complex binding site, which attracts I_3_^−^ by electrostatic force as well [37]. In addition to the electrostatic force, there is also hydrogen bonding interaction (N–H•••I) between the pyrrole-type N–H group and iodine species [38,39]. The above-mentioned I_3_^−^ adsorption modes and the inevitable pore capture belong to the category of physisorption, laying the foundation for the recycling of 7AIn-PiP. Moreover, the adsorbed I_3_^−^ (Lewis base) can interact with I_2_ (Lewis acid) through electron transfer to form I_5_^−^ in the skeleton [32], further promoting the enrichment of I_2_. The discovery of I_5_^−^ indicates that chemisorption also contributes to the enrichment process of the guest iodine species. To sum up, the I_2_ adsorption process of 7AIn-PiP in saturated I_2_ aqueous solution is affected by both physisorption and chemisorption. The corresponding schematic representation is depicted in Scheme 4. 

#### 3.2.5. Regeneration Property of 7AIn-PiP in Saturated I_2_ Aqueous Solution

**Figure 9:**
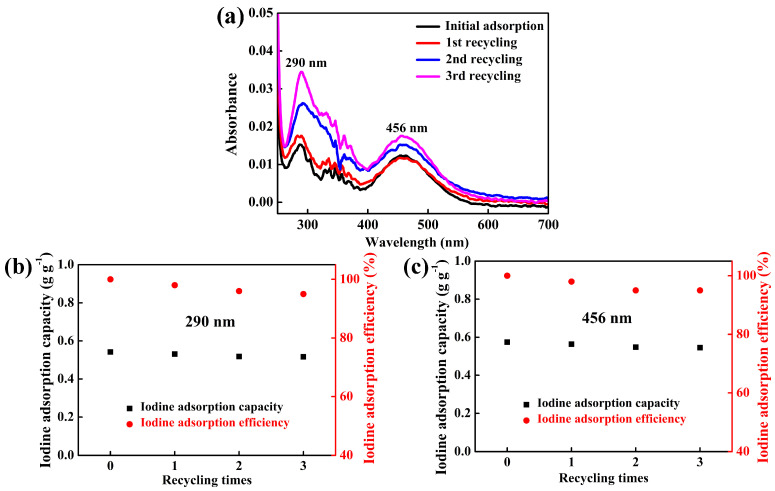
(**a**) UV–Vis absorption spectra of saturated I_2_ aqueous solutions after adsorption by 7AIn-PiP during recycling tests. Corresponding iodine adsorption capacity and iodine adsorption efficiency at (**b**) 290 and (**c**) 456 nm, respectively.

As seen in Figure 9a, the UV–Vis absorption curves of I_2_ aqueous solutions are no longer smooth, and the characteristic peaks shift slightly after adsorption. In particular, the characteristic peak at 354 nm is almost impossible to identify. Herein, 290 and 456 nm were chosen as the characteristic absorption wavelengths for the calculation of I_2_ adsorption capacity. As shown in Figure 9b,c and Table 3, 7AIn-PiP can still maintain about 95% of its original I_2_ adsorption efficiency after three rounds of recycling tests, indicating that our prepared adsorbent 7AIn-PiP indeed possesses excellent recycling ability and exhibits good development prospect in the field of I_2_ adsorption from water. 

#### 3.2.6. New Adsorption Phenomenon of 7AIn-PiP in Unsaturated I_2_ Aqueous Solution 

**Figure 10:**
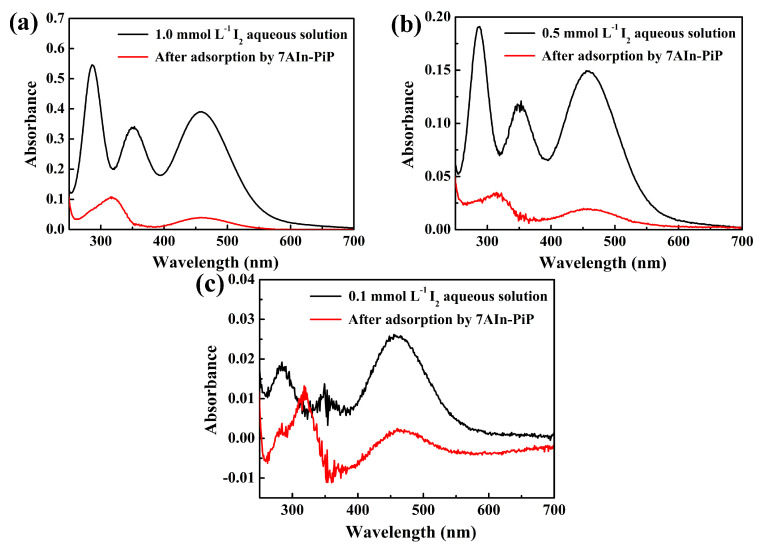
UV–Vis absorption spectra of I_2_ aqueous solutions with different concentrations [(**a**) 1.0, (**b**) 0.5, and (**c**) 0.1 mmol L^−1^] before and after adsorption by polymer 7AIn-PiP.

As shown in Figure 10a, it was found that when the initial concentration of I_2_ aqueous solution was reduced to 1.0 mmol L^−1^, the characteristic peaks at 290 and 354 nm disappear after adsorption by 7AIn-PiP, while a new peak at around 320 nm appears. In addition, when the initial concentration of I_2_ aqueous solution was further reduced to 0.5 and 0.1 mmol L^−1^, this phenomenon still exists (Figure 10b,c).

**Figure 11:**
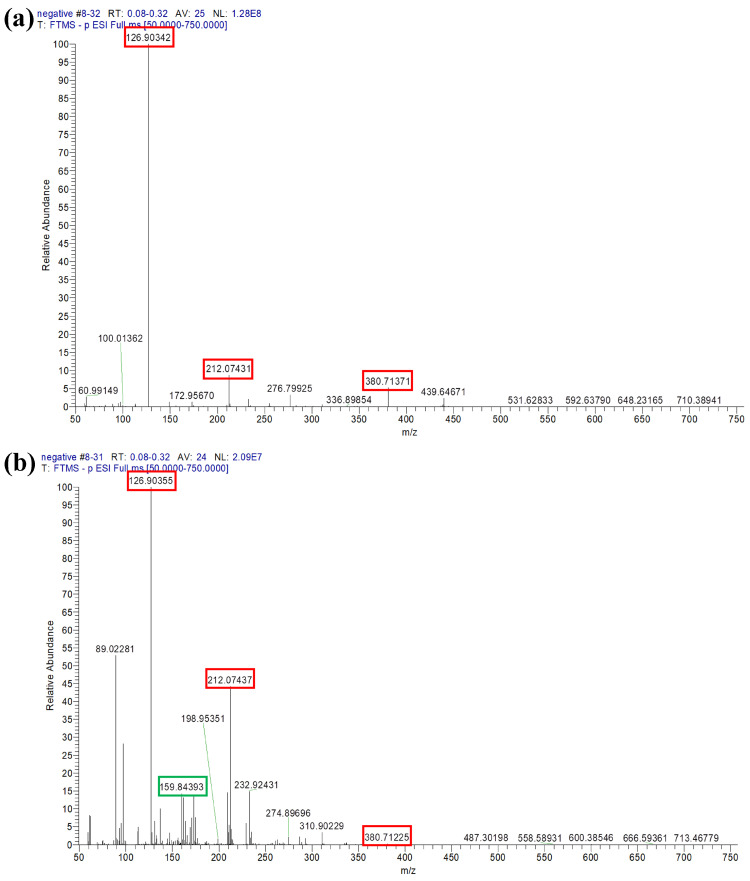
HRMS spectra of I_2_ aqueous solution (0.5 mmol L^−1^, 10.0 mL) before (**a**) and after (**b**) adsorption by 5.0 mg of 7AIn-PiP (the red rectangles represent the same peaks before and after adsorption, and the green rectangle represents the new peak after adsorption).

In order to reveal this interesting phenomenon, 0.5 mmol L^−1^ of I_2_ aqueous solutions before and after adsorption by 7AIn-PiP were characterized by HRMS with the negative-ion mode. As shown in Figure 11a,b, three characteristic peaks with a mass-to-charge ratio (*m*/*z*) of 126.90342/126.90355, 212.07431/212.07437, and 380.71371/380.71225 appear in the HRMS spectra of I_2_ aqueous solutions before and after adsorption. Of these, the peaks at 126.90342/126.90355 and 380.71371/380.71225 correspond to I^−^ (Exact mass: 126.90502) and I_3_^−^ (Exact mass: 380.71395), respectively, while the peak at 212.07431/212.07437 may be attributed to the form of HIO_3_•2H_2_O (Exact mass: 211.91817). After adsorption, the peak of I_3_^−^ is obviously weakened and hardly observed, indicating that I_3_^−^ is effectively adsorbed. Meanwhile, a new peak with a mass-to-charge ratio (*m*/*z*) of 159.84393 appears, corresponding to the newly generated HIO_2_ (exact mass:159.90212, weak acid, stable in water).

The detailed analysis is as follows: in the preparation of I_2_ aqueous solution, I_2_ and H_2_O weakly react to form HI and HIO [Equation (1)] [40,41]. As a result, HI completely dissociates to H^+^ and I^−^, while HIO is extremely unstable in water and is easily disproportionated to form I^−^ and IO_3_^−^ [Equations (2) and (3)] [40,41,42,43]. The generated I^−^ further reacts with molecular I_2_ to form I_3_^−^ [Equations (4) and (5)] [41,42,43,44], thus promoting the dissolution of solid I_2_. The associated ionic reaction equations are as follows:

I_2_ + H_2_O ⇌ I^−^ + HOI + H^+^ (1)

3HOI ⇌ 2I^−^ + IO_3_^−^ + 3H^+^ (2)

(1) × 3 + (2): 3I_2_ + 3H_2_O ⇌ 5I^−^ + IO_3_^−^ + 6H^+^ (3)

I_2_ + I^−^ ⇌ I_3_^−^ (4)

(4) × 3 − (3): IO_3_^−^ + 8I^−^ + 6H^+^ ⇌ 3I_3_^−^ + 3H_2_O (5)

When 7AIn-PiP is added into the I_2_ aqueous solution, a large number of I_3_^−^ are adsorbed rapidly through the electrostatic interaction generated by the cationic pyridinium adsorption sites, which shifts the equilibrium [Equation (5)] to the right and triggers the comproportionation between IO_3_^−^ and I^−^, inducing the formation of HIO_2_ in the aqueous solution [Equations (6)–(8)] [44]. The associated ionic reaction equations are as follows:

IO_3_^−^ + I^−^ + 2H^+^ ⇌ HOI + HIO_2_ (6)

2HOI ⇌ HIO_2_ + H^+^ + I^−^ (7)

(6) × 2 + (7): 2IO_3_^−^ + I^−^ + 3H^+^ ⇌ 3HIO_2_ (8)

## 4. Conclusions

In conclusion, a novel zwitterionic hypercrosslinked polymer (7AIn-PiP), featuring a unique dual 1,3-dipole composition, was successfully synthesized via one-pot S_N_2 nucleophilic substitution and FeCl_3_-catalyzed Friedel–Crafts alkylation reactions. In contrast to the previously reported ionic porous materials as iodine adsorbents from water [11,12,13], there is no exchangeable counterion in the skeleton of 7AIn-PiP during the iodine adsorption process. 7AIn-PiP possesses a high I_2_ removal rate (94.2%, at 456 nm) and fast adsorption kinetics (reaching adsorption equilibrium in 30 min) from the saturated I_2_ aqueous solution, mainly resulting from the synergistic effect of the constructed multiple binding sites. Moreover, the adsorption kinetic experiments show that the I_2_ adsorption behavior of 7AIn-PiP is fitted with the pseudo-second-order kinetic model, and the adsorption rate is influenced by both liquid-film diffusion and intra-particle diffusion. The adsorption mechanism was further studied by XPS, Raman, and FTIR spectra of I_2_@7AIn-PiP, clarifying that the I_2_ adsorption process of 7AIn-PiP is influenced by both chemisorption and physisorption. In addition, 7AIn-PiP still maintains good I_2_ adsorption performance (~95% I_2_ removal rate) after three adsorption–regeneration cycles, which is favorable for tackling the problem of removing radioactive iodine from nuclear sewage. Furthermore, we discovered and explained that the rapid adsorption of I_3_^−^ by 7AIn-PiP in unsaturated I_2_ aqueous solution can induce the formation of HIO_2_. To sum up, this work provides a new strategy for the design and development of rare zwitterionic porous organic polymers and has a certain basic research significance for the removal of radioactive iodine from water.

## Figures and Tables

**Table 1:** Parameters of pseudo-second-order kinetic model at 290, 354, and 456 nm, respectively.

Characteristic Absorption Wavelength *λ*_max_ (nm)	Experimental Adsorption Capacity at Equilibrium *q*_e, exp_ (g g^−1^)	Parameters of Pseudo-Second-Order Model		
		*q*_e, cal_ (g g^−1^)	*k*_2_ (g g^−1^ min^−1^)	*R* ^2^
290	0.542	0.545	12.125	1.00000
354	0.543	0.546	12.506	0.99999
456	0.574	0.582	3.589	0.99996

**Table 2:** Parameters of intra-particle diffusion kinetic model at 290, 354, and 456 nm, respectively.

Characteristic Absorption Wavelength *λ*_max_ (nm)	The First Stage			The Second Stage		
	*k*_p_ (g g^−1^ min^−0.5^)	*C* (g g^−1^)	*R* ^2^	*k*_p_ (g g^−1^ min^−0.5^)	*C* (g g^−1^)	*R* ^2^
290	0.01382	0.49546	0.99947	0.00172	0.53318	0.90717
354	0.01164	0.50157	0.95437	0.00143	0.53513	0.93978
456	0.02988	0.46206	0.94966	0.00703	0.5354	0.95255

**Table 3:** Iodine adsorption capacity and iodine adsorption efficiency of 7AIn-PiP during recycling tests in saturated I_2_ aqueous solution.

Adsorbent	The Mass of Adsorbent (mg)	Iodine Adsorption Capacity of 1 g Adsorbent (g g^−1^)		Iodine Adsorption Efficiency (%)	
		290 nm	456 nm	290 nm	456 nm
Original 7AIn-PiP	5.0	0.542	0.574	100	100
After the 1st regeneration	5.1	0.531	0.563	98.0	98.1
After the 2nd regeneration	5.2	0.518	0.548	95.6	95.5
After the 3rd regeneration	5.2	0.516	0.545	95.2	94.9

## Data Availability

The original contributions presented in the study are included in the article and Supplementary Material.

## Supplementary Materials

The following supporting information can be downloaded at https://www.mdpi.com/article/10.3390/polym16192846/s1. Figure S1. (a) UV–Vis absorption spectra of I_2_ aqueous solutions with different concentrations. (b) Corresponding standard curves at 290, 354, and 456 nm, respectively. Table S1. Yields and pore properties of polymer-1, -2, -3, -4, -5, and -6. Figure S2. (a) UV–Vis absorption spectra of I_2_ aqueous solutions before and after adsorption by a batch of obtained polymers under the same conditions. Corresponding I_2_ adsorption capacity and removal rate at (b) 290, (c) 354, and (d) 456 nm, respectively. Figure S3. FTIR spectrum of the dual 1,3-dipole. Table S2. Analysis of characteristic peaks of the dual 1,3-dipole. Table S3. Summary of I_2_ removal rates of various adsorbents from I_2_ aqueous solutions. References [45,46,47,48,49,50,51,52,53,54,55,56,57,58,59] are cited in the Supplementary Materials.
