# Supplementary material for: Synthesis of a Novel Zwitterionic Hypercrosslinked Polymer for Highly Efficient Iodine Capture from Water
Source: Polymers (Basel). 2024 Oct 9;16(19):2846. doi: 10.3390/polym16192846 (PMC11478924; doi:10.3390/polym16192846)
Supplement: Supplementary file 1 [file polymers-16-02846-s001.zip › polymers-3127560-supplementary.pdf]

Supplementary Materials

Synthesis of a novel zwitterionic hypercrosslinked polymer for highly efficient iodine capture from water

Jingwen Yu [1,2], Luna Song [3], Bingying Han [1], Jiangliang Hu [1], Zhong Li [1,*], Jie Mi [1,*]

[1] State Key Laboratory of Clean and Efficient Coal Utilization/Key Laboratory of Coal Science and Technology of Shanxi Province and Ministry of Education, Taiyuan University of Technology, Taiyuan 030024, China; 17836204253@163.com (J.Y.); hanbingying@tyut.edu.cn (B.H.); hujiangliang@tyut.edu.cn (J.H.)

[2] Lu′an Chemical Group Co., Ltd., Changzhi 046204, China

[3] Shanxi Institute of Energy, Jinzhong 030600, China; sxdxsln@163.com

* Correspondence: lizhong@tyut.edu.cn (Z.L.); mijie111@163.com (J.M.)

Contents

S1. Preparation and characterization of I_2 aqueous solutions 2

1.1. Preparation of a series of I_2 standard aqueous solutions 2

1.2. Characteristic absorption wavelengths of I_2 aqueous solutions and corresponding standard curves 2

S2. Preparation of a batch of polymers and screening of the optimal adsorbent 2

2.1. Preparation of a batch of polymers 2

2.2. Screening of the optimal adsorbent 3

S3. Adsorption kinetics of 7AIn-PiP in saturated I_2 aqueous solution 4

S4. FTIR spectrum and characteristic peaks analysis of the dual 1,3-dipole 5

S5. Summary of I_2 removal rates of different materials in I_2 aqueous solutions at different characteristic absorption wavelengths 5

S6. References 7

S1. Preparation and characterization of I_2 aqueous solutions

1.1. Preparation of a series of I_2 standard aqueous solutions

Firstly, 152.3 mg of I_2 was completely dissolved in 500.0 mL of deionized water to prepare saturated I_2 aqueous solution (1.2 mmol L^{-1}). Then, 10.0, 20.0, 30.0, 40.0, 50.0, 60.0, and 70.0 mL of stock solutions were diluted to volume of 100.0 mL by deionized water to get a series of I_2 standard aqueous solutions with different concentrations (0.12, 0.24, 0.36, 0.48, 0.60, 0.72, and 0.84 mmol L^{-1}), which were measured by UV–Vis absorption spectra. Accordingly, the standard curves at corresponding characteristic absorption wavelengths were obtained.

1.2. Characteristic absorption wavelengths of I_2 aqueous solutions and corresponding standard curves

UV–Vis absorption spectra of I_2 aqueous solutions with different concentrations (Figure S1a) and corresponding standard curves at different characteristic absorption wavelengths (Figure S1b) are the same as that previously reported by our group [1]. We used them as uniform standards for calculating the I_2 adsorption capacity and I_2 removal rate of a series of indole-based porous organic polymers. As shown in Figure S1a, the characteristic absorption wavelengths of I_2 aqueous solution are determined to be 290, 354, and 456 nm, respectively. The UV–Vis spectra show strong I_3^- peaks (290 and 354 nm), indicating that molecular I_2 tends to form I_3^- in the aqueous phase [2–4].

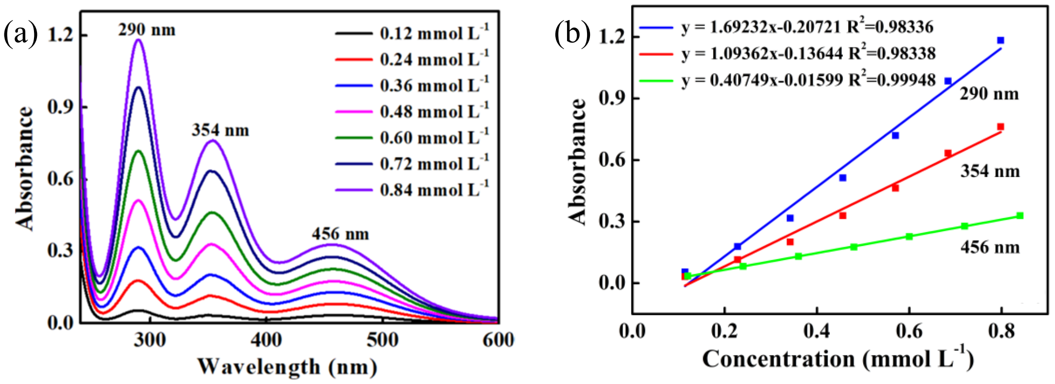

Figure S1. (**a**) UV–Vis absorption spectra of I_2 aqueous solutions with different concentrations. (**b**) Corresponding standard curves at 290, 354, and 456 nm, respectively [1].

S2. Preparation of a batch of polymers and screening of the optimal adsorbent

2.1. Preparation of a batch of polymers

Keeping the dosages of DCX and anhydrous $FeCl_3$ unchanged, the pore properties of polymers were adjusted by altering the molar ratio of 7AIn to DCX. Under N_2 atmosphere, a certain amount of 7AIn (Table S1) and DCX (1.7506 g, 10 mmol) were added into a dry 100 mL double-necked round-bottomed flask equipped with a reflux condenser and a magnetic stirrer. After 60 mL of DCE was injected into the flask under N_2 protection, the mixture was stirred at 80 °C for 24 h. After the reaction system was cooled to room temperature, anhydrous $FeCl_3$ (1.6220 g, 10 mmol, the molar ratio of $FeCl_3$ to DCX was kept 1:1 [5,6]) was added to the mixture under N_2 protection as soon as possible. Then, the obtained mixture was refluxed at 80 °C for an additional 24 h. After being cooled down to ambient temperature, the insoluble solid was obtained by suction filtration and continuously washed with methanol to remove the unreacted monomers, oligomers, and $FeCl_3$ until the filtrate

became colorless. The material was further purified by Soxhlet extraction with methanol for 24 h and then vacuum-dried at 90 °C for another 24 h to obtain the corresponding yellow-brown powder. The yield is calculated as formula (**1**).

$$\text{Yield (\%)} = \frac{\text{The mass of polymer}}{\text{The mass of total monomers} - \text{The mass of generated HCl}} \times 100\% \quad (\mathbf{1})$$

The obtained polymers with different 7AIn/DCX molar ratios of 1∶2, 1∶4, 1∶6, 1∶8, 1∶10, and the self-condensation of DCX (no added 7AIn) are referred to as Polymer-1, -2, -3, -4, -5, and -6, respectively. Their pore properties were characterized by N_2 adsorption-desorption experiments.

Table S1. Yields and pore properties of polymer-1, -2, -3, -4, -5, and -6.

Entry	Molar ratio of 7AIn to DCX	Dosage of 7AIn	Material name and yield	Specific surface area (S_{BET}, $m^2\ g^{-1}$)	Total pore volume (V, $cm^3\ g^{-1}$)	Average pore size (nm)
1	1∶2	590.7 mg (5 mmol)	Polymer-1 (838.5 mg, 52%)	14	0.045	12.839
2	1∶4	295.4 mg (2.5 mmol)	Polymer-2 (1.2483 g, 95%)	402	0.334	3.320
3	1∶6	197.3 mg (1.67 mmol)	Polymer-3 (1.1785 g, 97%)	622	0.475	3.055
4	1∶8	147.7 mg (1.25 mmol)	Polymer-4 (1.1235 g, 96%)	756	0.527	2.786
5	1∶10	118.1 mg (1 mmol)	Polymer-5 (1.1078 g, 97%)	814	0.614	3.018
6	Self-condensation of DCX (no added 7AIn)	0	Polymer-6 (999.8 mg, 98%)	1411	1.593	4.518

2.2. Screening of the optimal adsorbent

In order to screen the optimal adsorbent, the I_2 adsorption properties of these polymers in saturated I_2 aqueous solution were investigated. All materials participated in the adsorption procedure under the same conditions: at room temperature, 5.0 mg of adsorbent was added to a saturated I_2 aqueous solution (1.2 mmol L^{-1}, 10 mL) and stirred at a speed of 500 rpm. Then, the supernatant was gauged by employing UV–Vis spectroscopy until adsorption saturation was reached (until 30 min). The best performing material Polymer-2 was renamed as 7AIn-PiP. The I_2 removal rate and I_2 adsorption capacity are calculated according to formulas (**2**) and (**3**), respectively.

$$\text{Removal rate (\%)} = \frac{c_0 - c_t}{c_0} \times 100\% \quad (\mathbf{2})$$

$$q_t = \frac{(c_0 - c_t) \times M \times V}{m \times 1000} \quad (\mathbf{3})$$

where c_0 (1.2 mmol L^{-1}) and c_t (mmol L^{-1}) represent the concentration of I_2 aqueous solution before and after adsorption; q_t (g g^{-1}) is the I_2 adsorption capacity at time t (min); M (253.81 g mol^{-1}) is the molecular weight of I_2; V (10 mL) is the volume of I_2 aqueous solution; and m (5.0 mg) is the mass of adsorbent. The experimental results are shown in the Figure S2.

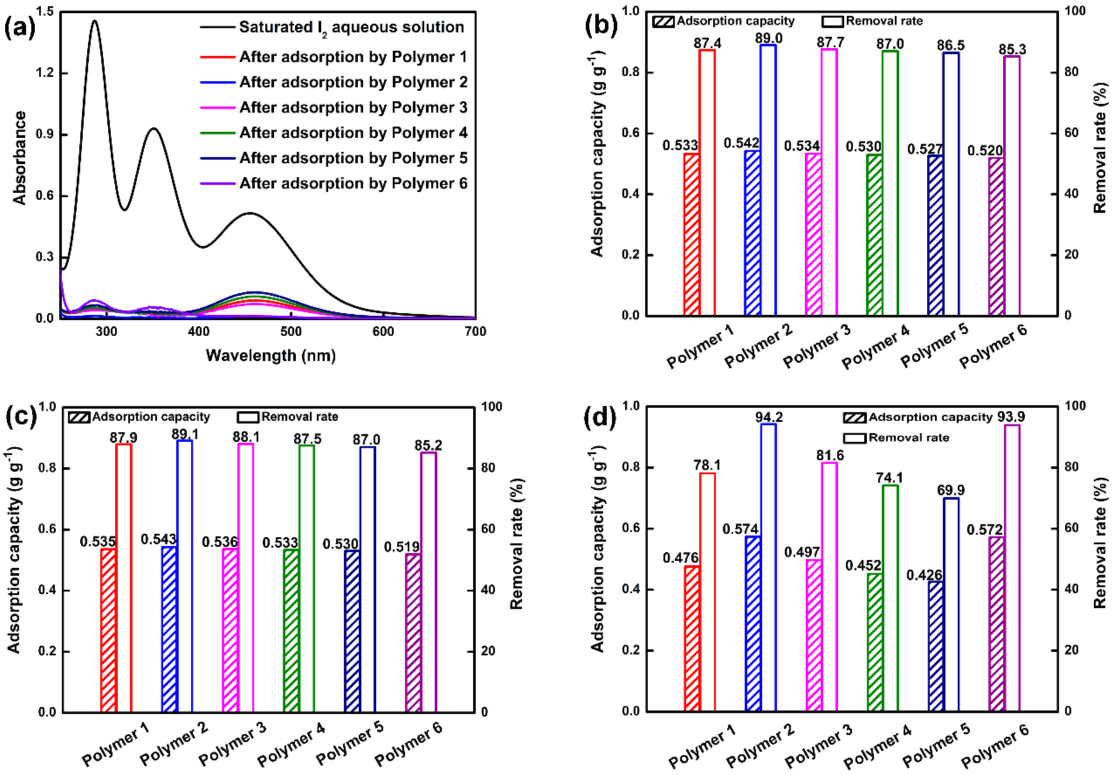

Figure S2. (**a**) UV–Vis absorption spectra of I_2 aqueous solutions before and after adsorption by a batch of obtained polymers under the same conditions. Corresponding I_2 adsorption capacity and removal rate at (**b**) 290, (**c**) 354, and (**d**) 456 nm, respectively.

S3. Adsorption kinetics of 7AIn-PiP in saturated I_2 aqueous solution

In order to monitor the I_2 capture speed of 7AIn-PiP in saturated I_2 aqueous solution, the experiment was performed as follows: at room temperature, 5.0 mg of 7AIn-PiP was added to a saturated I_2 aqueous solution (1.2 mmol L^{-1}, 10 mL) and stirred at a speed of 500 rpm. The UV–Vis spectrum of the solution was recorded per 3 min until adsorption equilibrium was reached (2 mL of supernatant withdrawed from the mother solution should be transferred back when each measurement was finished).

In order to describe the kinetic adsorption behaviors of 7AIn-PiP, the most widely used pseudo-second-order [Linear equation (**4**)] and intra-particle diffusion [Linear equation (**5**)] kinetic models were applied to fit the experimental data. The linear equations of these two models can be written as follows:

$$\frac{t}{q_t} = \frac{1}{k_2 \times q_{e,cal}^2} + \frac{t}{q_{e,cal}} \qquad (\mathbf{4})$$

$$q_t = k_p \times t^{0.5} + C \qquad (\mathbf{5})$$

where t (min) is the contact time; q_t (g g^{-1}) and $q_{e,cal}$ (g g^{-1}) are the adsorption capacity at time t and at equilibrium, respectively; k_2 (g g^{-1} min^{-1}) and k_p (g g^{-1} $min^{-0.5}$) are the adsorption rate constants for the pseudo-second-order and intra-particle diffusion kinetic models, respectively; C (g g^{-1}) is the intercept related to the thickness of the boundary layer.

For pseudo-second-order model, the values of $q_{e,cal}$ and k_2 are determined respectively by the slope ($1/q_{e,cal}$) and intercept [$1/(k_2 \times q^2_{e,cal})$] of the linear plots of (t/q_t) versus t; for intra-particle diffusion model, the values of k_p and C are calculated respectively from the slope and intercept of

the linear plots of q_t versus $t^{0.5}$.

S4. FTIR spectrum and characteristic peaks analysis of the dual 1,3-dipole

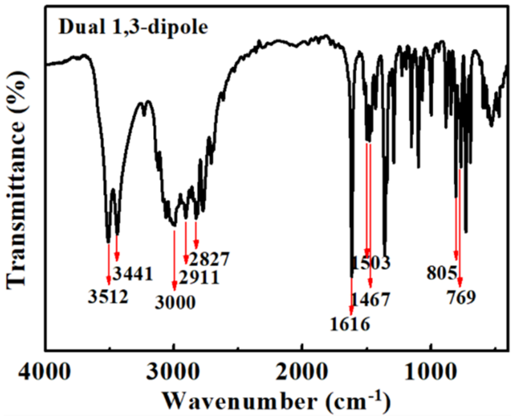

Figure S3. FTIR spectrum of the dual 1,3-dipole.

Table S2. Analysis of characteristic peaks of the dual 1,3-dipole.

Wave number /cm^{-1}	Vibrational mode of characteristic group
3512	O–H stretching vibration of residual methanol
3441	O–H stretching vibration of water coming from KBr
3000	C–H stretching vibration of aromatic ring (7AIn ring and benzene ring)
2911, 2827	C–H stretching vibration of methylene
1616, 1503	Skeleton stretching vibration of aromatic ring (7AIn ring and benzene ring)
1467	C–H bending vibration of methylene
805	C–H bending vibration of two adjacent hydrogen atoms on 7AIn ring/benzene ring
769	C–H bending vibration of three adjacent hydrogen atoms on 7AIn ring

S5. Summary of I_2 removal rates of different materials in I_2 aqueous solutions at different characteristic absorption wavelengths

The I_2 removal rates of different materials under similar test conditions are summarized in Table S3. Although the I_2 removal rates of 7AIn-PiP is lower than some materials such as metal-organic cages (PyrC and NapC), supramolecular organogel (G-TP5), covalent triazine framework (*MeO*-CTF600), hydrogen-bonded cross-linked organic framework (H_COF-1), aniline-based macrocyclic arene (An[2]s), macrocycle polymeric networks (C[4]P-TEPM and C[4]P-HEPM) and nitrogen-rich covalent organic framework (TTPA-COF), it is comparable to some adsorbents such as nonporous amorphous electron-deficient cyclophane (BPy-Box·4PF_6), pillar[5]arene-based 3D polymer network (DTTP5), and I3CA-POP. Furthermore, the I_2 removal rate of 7AIn-PiP is higher than cucurbit[8]uril-based supramolecular framework material (Q[8]-(4-AP)), some charge-neutral porous organic polymers (ITN-POP, TN-POP, TAT-POP, and Isatin-POP), censer-shaped macrocycle (CTX[P(O)Ph]), and even commercial AC. It follows from the above that 7AIn-PiP still exhibits excellent adsorptive property for I_2 from water.

Table S3. Summary of I_2 removal rates of various adsorbents from I_2 aqueous solutions.

Type and name of adsorbent		Dosage of adsorbent	Concentration and volume of I_2 aqueous solution	Adsorption time	I_2 removal rate	Ref.
Metal-organic cages	PyrC	5.0 mg	1.2 mmol L^{-1}, 50 mL	60 min	99.0% (461 nm)	7
	NapC	5.0 mg	1.2 mmol L^{-1}, 50 mL	60 min	99.6% (461 nm)	
Covalent triazine framework	*MeO*-CTF600	10.0 mg	1.63 mmol L^{-1}, 10 mL	55 s	>98% (286/350 nm)	8
Censer-shaped macrocycle	CTX[P(O)Ph]	20.0 mg	4 mL	4 h	90% (460 nm)	9
Aniline-based macrocyclic arene	An[2]s	5.0 mg	1.0 mmol L^{-1}, 3 mL	2 min	99.9% (460 nm)	10
Nitrogen-rich covalent organic framework	TTPA-COF	10.0 mg	1.2 mmol L^{-1}, 10 mL	15 min	98.7% (~ 460 nm)	11
Cucurbit[8]uril-based supramolecular framework material	Q[8]-(4-AP)	20.0 mg	1.2 mmol L^{-1}, 5 mL	80 min	91% (458 nm)	12
Nonporous amorphous electron-deficient cyclophane	Bpy-Box·4PF_6	3.0 mg	1.2 mmol L^{-1}, 3 mL	300 min	>90% (~ 460 nm)	13
Supramolecular organogel	G-TP5	3.0 mg	1.0 mmol L^{-1}, 3 mL	60 min	96.1% (286/350 nm)	14
Hydrogen-bonded cross-linked organic framework	HcOF-1	3.0 mg	1.14 mmol L^{-1}, 2 mL	30 min	93.8% (286/350 nm)	15
				4 h	97.2% (286/350 nm)	
Macrocycle polymeric networks	C[4]P-TEPM	2.5 mg	1.2 mmol L^{-1}, 2.5 mL	4 min	95.0% (460 nm)	16
	C[4]P-HEPM	2.5 mg	1.2 mmol L^{-1}, 2.5 mL	4 min	97.6% (460 nm)	
Pillar[5]arene-based 3D polymer network	DTTP5	3.0 mg	0.1 mg mL^{-1}, 3 mL	30 min	90.9% (286 nm)	17
Porous organic polymers	ITN-POP	10.0 mg	1.14 mmol L^{-1}, 5 mL	5 h	91.6% (461 nm)	18
	TN-POP	10.0 mg	1.14 mmol L^{-1}, 5 mL	5 h	75.9% (461 nm)	
	I3CA-POP	5.0 mg	1.2 mmol L^{-1}, 10 mL	30 min	87.3% (290 nm)	1
					87.0% (354 nm)	
					96.1% (456 nm)	
	TAT-POP	5.0 mg	1.2 mmol L^{-1}, 10 mL	30 min	73.0% (290 nm)	
					72.7% (354 nm)	
					89.9% (456 nm)	
	Isatin-POP	5.0 mg	1.2 mmol L^{-1}, 10 mL	30 min	65.9% (290 nm)	
					65.4% (354 nm)	
					87.9% (456 nm)	
Commercial activated carbon	AC	5.0 mg	1.2 mmol L^{-1}, 10 mL	30 min	78.7% (290 nm)	
					78.2% (354 nm)	
					91.6% (456 nm)	
Zwitterionic hypercrosslinked polymer	7AIn-PiP	5.0 mg	1.2 mmol L^{-1}, 10 mL	30 min	89.0% (290 nm)	This work
					89.1% (354 nm)	
					94.2% (456 nm)	

S6. References

1. Yu, J.W.; Song, L.N.; Wang, Y.S.; Bai, T.H.; Long, C.M.; Wu, M.M.; Feng, Y.; Mi, J. Three novel indole-based porous organic polymers for efficient iodine capture in water. *J. Radioanal. Nucl. Ch.* **2023**, *332*, 4271–4290.
2. Zhou, W.; Li, A.M.; Zhou, M.; Xu, Y.Y.; Zhang, Y.; He, Q. Nonporous amorphous super adsorbents for highly effective and selective adsorption of iodine in water. *Nat. Commun.* **2023**, *14*, 5388.
3. Avais, M.; Chattopadhyay, S. Porous polyaminoamides *via* an exotemplate synthesis approach for ultrahigh multimedia iodine adsorption. *J. Mater. Chem. A*. **2022**, *10*, 20090−20100.
4. Sen, A.; Sharma, S.; Dutta, S.; Shirolkar, M.M.; Dam, G.K.; Let, S.; Ghosh, S.K. Functionalized ionic porous organic polymers exhibiting high iodine uptake from both the vapor and aqueous medium. *ACS Appl. Mater. Interfaces*. **2021**, *13*, 34188− 34196.
5. Xiong, S.H.; Tang, X.; Pan, C.Y.; Li, L.; Tang, J.T.; Yu, G.P. Carbazole-bearing porous organic polymers with a mulberry-like morphology for efficient iodine capture. *ACS Appl. Mater. Interfaces*. **2019**, *11*, 27335−27342.
6. Chen, D.Y.; Fu, Y.; Yu, W.G.; Yu, G.P.; Pan, C.Y. Versatile adamantane-based porous polymers with enhanced microporosity for efficient CO_2 capture and iodine removal. *Chem. Eng. J.* **2018**, *334*, 900−906.
7. Zeng, Z.Y.; Lou, Z.C.; Hu, L.R.; Dou, W.T.; Zhao, X.L.; Li, X.D.; Fang, J.F.; Qian, X.H.; Yang, H.B.; Xu, L. Metal-organic cages for efficient capture and convenient detection of iodine from vapor and aqueous solutions. *Chem. Eng. J.* **2024**, *496*, 154091.
8. Das, M.; Sarkar, S.K.; Patra, Y.S.; Manna, A.; Mukherjee, S.; Das, S. Soft self-templating approach-derived covalent triazine framework with bimodal nanoporosity for efficient radioactive iodine capture for safe nuclear energy. *ACS Appl. Nano Mater.* **2022**, *5*, 8783− 8793.
9. Jiao, J.M.; Xie, W.; Li, Y.M.; Lin, C.; Jiang, J.L.; Wang, L.Y. Effective iodine capture behavior by a censer-shaped macrocycle in vapor phase and aqueous solution. *Chem. Eur. J.* **2022**, *28*, e202201933.
10. Jin, P.Y.; Liang, W.T.; Rong, Y.Q.; Wu, W.A.H.; Gou, M.; Tang, Y.Q.; Yang, C. Remarkable iodine uptake by aniline-based macrocyclic arenes through a reverse dissolution mechanism. *J. Mater. Chem. A*. **2023**, *11*, 11126−11132.
11. Shreeraj, G.; Sah, A.; Sarkar, S.; Giri, A.; Sahoo, A.; Patra, A. Structural modulation of nitrogen-rich covalent organic frameworks for iodine capture. *Langmuir*. **2023**, *39*, 16069− 16078.
12. Lu, Y.; Yu, Z.C.; Zhang, T.T.; Pan, D.W.; Dai, J.J.; Li, Q.; Tao, Z.; Xiao, X. A cucurbit[8]uril - based supramolecular framework material for reversible iodine capture in the vapor phase and solution. *Small*. **2024**, *20*, 2308175.

13. Wu, B.Q.; Li, Z.W.; Lin, F.; Tang, R.Z.; Zhang, W.Q.; Liu, H.W.; Ouyang, G.F.; Tan, Y. The paradigm for exceptional iodine capture by nonporous amorphous electron-deficient cyclophanes. *J. Hazard. Mater.* **2024**, *465*, 133449.
14. Li, B.; Wang, B.; Huang, X.Y.; Dai, L.; Cui, L.; Li, J.; Jia, X.S.; Li, C.J. Terphen[n]arenes and quaterphen[n]arenes (n=3-6): one-pot synthesis, self-assembly into supramolecular gels, and iodine capture. *Angew. Chem. Int. Ed.* **2019**, *58*, 3885−3889.
15. Lin, Y.X.; Jiang, X.F.; Kim, S.T.; Alahakoon, S.B.; Hou, X.S.; Zhang, Z.Y.; Thompson, C.M.; Smaldone, R.A.; Ke, C.F. An elastic hydrogen-bonded cross-linked organic framework for effective iodine capture in water. *J. Am. Chem. Soc.* **2017**, *139*, 7172−7175.
16. Zheng, Z.Y.; Lin, Q.Y.; Xie, L.H.; Chen, X.L.; Zhou, H.; Lin, K.H.; Zhang, D.S.; Chi, X.D.; Sessler, J.L.; Wang, H.Y. Macrocycle polymeric networks based on a chair-like calix[4]pyrrole for the rapid and efficient adsorption of iodine from water. *J. Mater. Chem. A*. **2023**, *11*, 13399−13408.
17. Cao, J.J.; Zhu, H.T.Z.; Shangguan, L.Q.; Liu, Y.Z.; Liu, P.R.; Li, Q.; Wu, Y.T.; Huang, F.H. A pillar[5]arene-based 3D polymer network for efficient iodine capture in aqueous solution. *Polym. Chem.* **2021**, *12*, 3517−3521.
18. Gao, R.; An, B.H.; Zhou, C.; Zhang, X. Synthesis of a triazaisotruxene-based porous organic polymer and its application in iodine capture. *Molecules,* **2022**, *27*, 8722.
